# Lipid nanoparticles (LNP) induce activation and maturation of antigen presenting cells in young and aged individuals

**DOI:** 10.21203/rs.3.rs-2199652/v1

**Published:** 2022-11-07

**Authors:** Jennifer Connors, David Joyner, Nathan Mege, Gina Cusimano, Matthew Bell, Jennifer Marcy, Bhavani Taramangalam, Paulo Lin, Ying Tam, Paulo Lin, Drew Weissman, Michele Kutzler, Mohamad-Gabriel Alameh, Elias Haddad

**Affiliations:** Drexel University; Drexel University; Tower Health; Drexel University; Drexel University; Drexel University; Drexel University; Acuitas Therapeutics; Acuitas Therapeutics; Acuitas Therapeutics; University of Pennsylvania; Drexel University; University of Pennsylvania; Drexel University

## Abstract

Despite the overwhelming success of mRNA-based vaccine in protecting against SARS-CoV-2 infection and reducing disease severity and hospitalization, little is known about the role lipid nanoparticles (LNP) play in initiating immune response. In this report we studied the adjuvantive impact of empty LNP with no mRNA cargo (eLNP) on anti-viral pathways and immune function of cells from young and aged individuals. We found that eLNP induced maturation of monocyte derived dendritic cells by measuring the expression of CD40, CD80, HLA-DR and production of cytokines including IFN-α,IL-6, IFN-γ, IL-12, and IL-21. Flow cytometry analysis of specific dendritic cell subsets showed that eLNP can induce CD40 expression and cytokine production in cDC1, cDC2 and monocytes. Empty LNP (eLNP) effects on dendritic cells and monocytes coincided with induction pIRF7 and pTBKI, which are both important in mitigating innate immune signaling. Interestingly our data show that in response to eLNP stimulus at 6 and 24 hrs, aged individuals have decreased CD40 expression and reduced IFN- γ output compared to young adults. Furthermore, we show that cDC1, cDC2, and CD14^dim^ CD16^+^ monocytes from healthy aged individuals have dysregulated anti-viral signaling response to eLNP stimulation as measured by the defect in type I IFN production, phosphorylation of IRF7, TBK-1, and immune function like phagocytosis. These data showed a novel function of eLNP in eliciting DC maturation and innate immune signaling pathways and that some of these functions are impaired in older individuals providing some suggestion of why older individuals (> 65 yrs of age) respond display lower immune responses and adverse events to SARS-CoV-2 mRNA-based vaccines.

## Introduction

Two of the current COVID-19 vaccine designs are based on nucleoside-modified messenger RNA (mRNA)-lipid nanoparticles (LNP). This vaccine platform has been widely tested in preclinical and clinical studies showing its effectiveness in generating protective humoral immunity^1, 2, 3, 4, 5^. Additionally, it has been shown that the immune stimulatory effects of the mRNA-LNP vaccine platform is not due to the inclusion of nucleoside modified mRNA but by the intrinsic adjuvant effect of the ionizable lipid, a major component of the LNP formulation, which on its own can induce an inflammatory response^1, 6^. However, the mechanism of this response has not been fully investigated. Previous studies seeking to elucidate the mechanism of these immunomodulatory agents has identified the involvement of different pathogen-associated molecular pattern (PAMP)-sensing receptors and pathways in mice^1, 6, 7^ but few, if any, studies exist in humans. Recently, Alameh et. al published that the addition of empty LNP (eLNP) not only intrinsically promoted the secretion of IL-6, among other cytokines in mice, but also induced robust T_FH_ cell responses after immunization. Removal of the ionizable lipid from the LNP abrogated the vaccine response highlighting its essential role in adjuvanticity. Another group recently published that lipid-formulated RNA vaccines induce production of IL-1 which then induce pro-inflammatory cytokines like IL-6^8^.

Although more studies are needed to determine how the LNP is sensed and how pro-inflammatory cytokines and chemokines including IL-6 production are regulated. Age is another factor that can influence vaccine response. The progressive decline in the function of the immune system with increasing age is a condition known as immunosenescence which not only leads to decreased protection against infectious pathogens but also leads to the inability to mount protective immune responses following vaccination^9^. An age associated decrease in protection against infectious pathogens is demonstrated by the increased mortality rates in individuals above 65 years of age following infection. An increase in mortality in aged individuals was clearly demonstrated during the COVID-19 pandemic where individuals aged 65–74 were upwards of 95 times more likely to die from SARS-CoV-2 infection when compared to the 18–29-year-old population^10^. With the global population over the age of 65 expected to double by the year 2050^11^, it is imperative to gain a deeper understanding of the underlying mechanisms of immunosenescence to effectively protect this portion of the population.

In those over 65 it has been shown that there are deficits in innate antiviral signaling and adaptive immune response to the SARS-CoV-2 vaccine particularly for variants of concern, suggesting that LNP stimulatory effects could be altered in the aged.^12^ Many of the recent studies that look at SARS-CoV-2 vaccination in people over 65 show induction of strong humoral responses against the virus, and its variants^13^. Other studies show that coordination of SARS-CoV-2 antigen specific responses are disrupted in those individuals^14^. The data suggests less of a role for the humoral response than SARS-CoV-2 specific CD4 + or CD8 + T cells in providing complete protection against severe disease in older adults. Understanding how LNP interacts with the innate immune system, especially within an aging context can help us to continue to develop adjuvants to induce stronger and more durable responses in older individuals.

In this report, we demonstrate that the eLNP formulation used in previous studies in mice^1, 2^ is effective at maturing monocyte-derived dendritic cells and activating DCs and monocytes from human PBMCs causing secretion of not only innate immune cytokines and chemokines but also pro-T_FH_ cytokines including IL-21 and IL-6^15^. We also demonstrate that there is an age-specific difference between important innate immune cytokines and chemokines and show that eLNP initiate TGF-β production as a potential mechanism of human T_FH_ cell differentiation^16^. Mechanistically, the capacity of eLNP to elicit robust induction of innate and adaptive responses shows evidence of being dependent on pathways that converge upon TBK-1 (phosphorylation of TBK-1) though differences between younger and older adults was less noticeable. We also show that the addition of eLNP acts as a stimulator of phagocytosis. This study sheds light on the mechanism of the immunomodulatory component of the recent SARS-CoV-2 vaccines and how we can improve vaccine efficacy in the more vulnerable population.

## Results

### eLNP treatment promotes robust innate immune response and DC maturation

The effect of eLNP on innate immune cells is not fully elucidated in humans. To investigate this, we tested the ability of eLNP to induce DC maturation in vitro. Monocytes isolated from healthy participant PBMCs (n=18; age range 24–75 yrs) were treated with GM-CSF/IL-4 for 48 hrs followed by maturation with a dose of 15 μg/mL (total lipids, or~7.5 μg/mL ionizable lipid) empty LNP (eLNP) for 24 hrs. We assessed the frequency of surface costimulatory and HLA marker-expressing cells in eLNP-treated MDDCs compared to unstimulated cells after 24 hrs. The gating strategy used for MDDCs, and the determination of their surface marker frequencies is shown in Supplemental Figure 1. We found that eLNP significantly up-regulated the percentage of CD40-positive human MDDCs compared to unstimulated control (p=0.0043) (Figure 1a). We also see an upregulation in the maturation markers of MDDCs (CD83 (p=0.0001), CD86 (p=0.0114), and HLA-DR (p=0.002) (Supplemental figure 2a and 2b). We also tested the production of cytokines following in vitro DC maturation by eLNP IL-6 (p=0.0321), IL-12 (p=0.0294), IL-21 (p<0.0001), CD40L (p=0.0009), IFNa (p=0.0040), and IFNg (p=0.0008) significantly increased after 24 hrs of eLNP stimulation (Figure 1b). Thus, eLNP can induce pro-T_FH_ cytokines as well as key cytokines and chemokines that are efficient in activating innate immune response like IFNa and IFNg. All together, these data using human MDDCs in our in vitro culture system confirm that eLNP can induce the maturation of human MDDCs and may a play a role in the initiation of innate immunity and a further role in T_FH_ differentiation, function, and proliferation^17^.

We further assess the impact of eLNP on multiple DC and monocyte subsets using multiparametric flow cytometry. Specifically, we determined the frequency of surface costimulatory marker-expressing cDC1, cDC2, and CD14 monocytes following eLNP treatment of PBMCs (Figure. 1c). To achieve this, PBMCs were stimulated with eLNP for 24 hrs and then DCs and monocytes subsets were analyzed for the expression of surface marker and cytokine secretion. Conventional type 1 DCs (cDC1) are the primary subset that cross-presents antigen to CD8+ T cells and predominantly produce IL-12^18, 19, 20, 21^. In contrast, conventional type 2 DCs (cDC2) have been associated with CD4+ T_FH_ cell responses including GC T_fh_ responses^17, 21, 22^. Additionally, we monitored CD14+CD16−, CD14+CD16+ and CD14^dim^CD16^+^ monocytes. The latter plays a vital role in antiviral immunity and response to vaccination by patrolling the vascular endothelium in response to viral exposure and produce TNF-a and IL-1b^23, 24^. We found that eLNP induces the expression of CD40 in cDC2 (p<0.0001) and cDC1 (p<0.0001), and CD14^dim^ CD16^+^ monocytes (p=0.0004). Additionally, we also see the upregulation of multiple activation markers on monocyte and DC subsets from PBMCS, namely OX40L (cDC2 (p<0.0001), cDC1 (p=0.0174)) (Supplemental figure 3b). We saw no significant changes in the other subsets of monocytes (data not shown). We also examined the eLNP-induced cytokine and chemokine profile of PBMCs. After stimulation with eLNP over 0-,6-, and -24 hrs, the production of cytokines and chemokines was significantly elevated consistent with a pro-inflammatory phenotype compared to unstimulated cells, namely IL-6 (p=<0.0001), IL-21 (p<0.0001), IFNg (p<0.0001), and CXCL13 (p=0.0001) (Figure 1d). To further investigate how eLNP might affect a first line defense, we isolated monocytes (CD14+) from PBMCs and stimulated with eLNP at 0-,6-, and -24 hrs and measured the production of cytokines and chemokines. We found that monocytes had significantly increased upregulation of IL-1b (p=0.0132), IL-6 (p=0.0208), and CX3CL1 (p=0.0040) by 6 hr that was sustained through 24 hrs (Figure 1e). The monocyte chemokines MIP1a and MCP-1 were significantly upregulated by 24 hrs post stimulation (MIP1α (p<0.0001) and MCP-1 (p<0.0001)). These data confirm that eLNP activates DCs and monocyte subsets and suggest the possibility that eLNP may mediate its effect by activation and maturation of innate immune cells.

### IRF7/TBK-1 axis is induced by eLNP

DCs and monocytes help shape the innate immune response via the activation of pattern recognition receptors (PRR) and other molecular sensors. The activation of different TLR (such as TLR 2,4, and 7/8) and RLR pathways initiates a series of cross-talk signaling events that are mediated by TBK-1, which leads to the phosphorylation, dimerization, and translocation of the transcription factor, IRF7^25, 26^. We next determined if eLNP can elicit phosphorylation and activation of IRF7 and TBK-1. Human PBMCs from 18 donors were stimulated with a dose of 15 μg/mL (total lipids, or~7.5 μg/mL ionizable lipid) eLNP for 15 min-, 45 min-, 6-, and 24 hrs, and flow cytometry was performed to determine whether eLNP alone can induce and activate IRF7 in DC and monocyte subsets, as measured by phosphorylation of IRF7 (pIRF7). Overall, eLNP treatment was able to notably induce IRF7 activation in cDC2 and cDC1 (Figure 2). Following a 6-hour stimulation, significant upregulation of pIRF7 was observed in cDC2 DCs (p= 0.0022) and was maintained through 24 hrs of stimulation (p= 0.0067) (Figure 2a). Significant upregulation of pIRF7 was observed as early as 45 min in cDC1 DCs (p= 0.0009), and this significant upregulation was enhanced and then maintained for both the 6- and 24-hour stimulations, respectively (6 hr p= <0.0001; 24 hr p= <0.0001). In addition to the phosphorylation of IRF7, we also saw phosphorylation of TBK-1 (pTBK-1) after eLNP stimulation in both cDC1 and cDC2 DCs (Figure 2b). Here, we saw a significant upregulation of pTBK-1 following a 6-hour stimulation in both cDC2 (p=0.0052) and cDC1(p<0.0001) DCs. These increased levels of pTBK-1 were maintained through a 24-hour stimulation for both DC subsets (cCD2, p= 0.0067; cDC1, p= <0.0001). We did not see a significant difference in pIRF7 or pTBK-1 induction in CD14^dim^ monocytes (Supplemental figure 4). Overall, these results demonstrate that eLNP can activate IRF and TBK-1 signaling pathways.

### Phagocytosis is induced in cDC2, cDC1 in response to eLNP stimulation.

A primary function of innate immune cells is phagocytosis. Here, we investigated whether eLNP can induce phagocytosis in antigen presenting cells utilizing an *in vitro* phagocytosis assay which measures the uptake of fluorescent beads. We stimulated healthy adult PBMCs with eLNP for 24 hrs and flow cytometry was performed to analyze the MFI of engulfed particles as a function of phagocytosis of DC subsets and CD14^dim^ monocytes. Upon stimulation with eLNP cDC2, cDC1 subsets show increased fluorescence (cDC2, p<0.0001; cDC1, p<0.0001) compared to unstimulated indicating superior phagocytic function following stimulation with eLNP (Figure 3a-b). CD14^dim^ monocytes show increased fluorescence when stimulated indicating greater activation (p<0.01) (Figure 3b).

### eLNP elicits TGF-β production in PBMCs and MDDCs

TGF-β has previously been shown to play essential roles in the differentiation and function of T cells, inhibiting the differentiation of Th1 and Th2 T cells^27^ while promoting Treg, Th17, and T_FH_ differentiation^27, 28^. To test whether eLNP treatment leads to production of TGF-β isoforms (TGF-β1, TGF-β2, and TGF-β3) within innate immune cells, PBMCs were treated for 6 or 24 hrs with eLNP (15 μg/μL-1 total lipids, or 7.5 μg/μL-1 ionizable lipid) and compared to both unstimulated cells. The levels of TGF-b1 in PBMCs were induced after 24 hrs of stimulation (p= <0.0001) while the levels of TGF-b3 were induced starting at 6 hrs (p= 0.0011) and increased when measured after 24 hrs of stimulation (p= <0.0001) (Figure 4a). Similarly, MDDCs from donors were treated for 24 hrs with eLNP and compared to unstimulated cells. Interestingly, TGFb2 levels were downregulated upon stimulation with eLNP for 24 hrs in MDDCs (p= 0.0065) suggesting a differential role between cell types (supplemental figure 5a).

### eLNP alter MDDC maturation and cytokine secretion in younger and older adults

After establishing that eLNP can induce an immune response, we aimed to describe how eLNP would affect an aged immune system. Therefore, we stratified our 18 subjects as young and old. Our old volunteers are healthy, non-frail individuals enrolled into the study are accrued with an equal sex distribution. Individuals with comorbid conditions like cancer within the last 5 years, or other immunocompromising conditions, and steroid use were excluded. Inclusion criteria included controlled hypertension, occasional aching joints from arthritis and not taking daily nonsteroidal anti-inflammatory drugs or acetaminophen, and controlled diabetes. The average age for adults was 30 years (range 24–36 years) whereas for older group it was 73 years (range 67–83 years) (Supplemental Table 1). Utilizing our cohort of 9 younger adults (<65) and 9 older adults (>65) we analyzed the effect of eLNP on the maturation and activation of the innate immune system. There were no significant differences in frequencies of cell types between eLNP stimulated cells from older or younger donors (data not shown). We found that eLNP significantly up-regulated the percentage of CD40− and CD83-positive human MDDC compared to unstimulated control (young, p=0.0079; old, p=0.0478) (Figure. 5a-d) for both older and younger participants. Specifically, the increase in eLNP-mediated CD83 expression was noted to be significantly different in older and younger participants (p= 0.0041) (Figure 5a). Empty LNP induced the expression of CD40 (Figure 5a) in both younger (p= 0.0006) and older participants, which is known to be important for human T_FH_ helper function via CD40L engagement. However, intergroup comparison showed that CD40 expression was higher for younger participants but did not reach statistical significance (p= 0.0939) (Figure 5a). We also examined the eLNP-induced cytokine and chemokine profile of MDDCs within this aged cohort. Empty LNP administration significantly increased the secretion of CD40L (young=0.0939, old p=0.0086), IL-2 (young=0.0015, old p=0.0031), and IL-12 (old p=0.0151) (Figure 5b). Namely, we saw significantly more cytokine production in older vs younger adults in CD40L (p=0.0061), IL-2 (p=0.0515), and IL12p70 (p=0.0376). Overall, our results show that eLNP can induce the maturation of MDDCs in vitro in both young and old individuals, however maturation markers are significantly lower in older subjects whereas pro-inflammatory cytokines are higher.

### eLNP induces age-specific activation in cDC1, cDC2, and CD14^dim^CD16^+^ monocytes from PBMCs

To determine the effect that eLNP has on the activation of monocyte and DC subsets between age groups, PBMCs from healthy young or older participant were treated with either eLNP for 6 or 24 hrs. We assessed the frequency of surface costimulatory marker-expressing cells in eLNP-treated PBMCs compared to unstimulated cells after 24 hrs (Figure 5c). 41BBL is expressed on antigen-presenting cells that, upon ligation to its cognate receptors, activates both CD4 and CD8 T cells and induces their proliferation^29, 30^. We found that that 41 BBL was upregulated with eLNP stimulation in cDC2 (p=0.0283) and cDC1 (p=0.0090) of younger donors. We also see an age specific difference between young and older participants in cDC2 when stimulated with eLNP (p=0.0019) and a trend in cDC1 (p=0.0977). We also observed a significant upregulation in the expression of CD40 due to eLNP stimulation in the same cell types cDC1 (young, p<0.0001; old, p=0.0004) and cDC2 (young, p<0.0001; old, p<0.0001). PD-L1 engagement limits the proliferation of antigen-specific T cells in the germinal center by binding to activated T cells, B cells, and other myeloid cells. We found that in response to eLNP stimulation, only cDC2 (p=0.0002), cDC1 (p=0.0007), and CD14^dim^ CD16^+^ monocytes (p=0.0055) from older donors upregulated PD-L1 (Figure 5c).

We also examined the eLNP-induced cytokine and chemokine profile of PBMCs. Empty LNP induced cytokine and chemokine profiles with elevated levels of expression after 24 hrs of stimulation specifically; IL-6 (young, p=0.0142; old, p=0.0076), IL-21 (young, p=0.0020; old, p=0.0030),IFNg (young, p<0.0001; old, p<0.0001),TNF-a (young, p=0.1069; old, p=0.0420), CXCL13 (young, p=0.0939; old, p=0.0025), CCL7 (young, p=0.0010; old, p=0.0078). Overall, these data suggest that eLNP is immunostimulatory even in older adults which combined with antigen can lead to a sufficient vaccine response.

### eLNP stimulation results in impairment of phagocytosis in CD14^dim^CD16^+^ monocytes in older adults

Upon stimulation with eLNP all subtypes of monocytes and DCs showed increased phagocytosis in both young and older adults (Figure 6a-b). Although eLNP enhanced the phagocytic ability of monocyte and DC subsets in all individuals, monocyte subtypes in aged individuals had reduced phagocytic activity (Figure 6b).

In DC subsets, there was no significant difference in phagocytosis between young and older adults in response to stimuli, but phagocytosis was induced in cDC2 (young: eLNP p=<0.0001; old: eLNP p= <0.0001) and cDC1 (young: eLNP p=<0.0001; old: eLNP p=<0.0001). Empty LNP was capable of inducing phagocytosis in younger adults in CD14^dim^ CD16^+^ with eLNP (p=0.0010). CD14^dim^ CD16^+^ from older adults had significant decrease in phagocytosis compared to younger adults when stimulated with eLNP (p=0.0106). Of note, cDC2 showed significant decrease in phagocytic ability in older adults in LPS/IFN-g levels (p=0.0091), which predicts that cDC2’s role in T cell differentiation and GC reaction might also significantly impaired in older adults (Supplemental figure 5a). This impairment was easily rescued when induced with eLNP and had similar amount of phagocytic function throughout the DC subsets, which further underscore the ability of eLNP to enhance phagocytosis.

### eLNP mediates differential production of TGF-β production between younger and older adults

To further understand the age differences following eLNP stimulation, TGF-β production by MDDCs and PBMCs from young and old participants was explored. For MDDCs, differences in TGF-β2 production were observed between young and old in immature cells (p=0.0232) (Figure 7a). However, following a 24-hour treatment with eLNP TGF-β2 production by MDDCs from young individuals was significantly lower than that found in older healthy adults (p=0.0005) (Figure 7a). Additionally, eLNP treatment led to a reduction in the production of all isoforms of TGF-β in aged individuals compared to levels detected from unstimulated MDDCs in the same aged individuals. In contrast, TGF-β production by MDDCs from young individuals was not found to be altered when compared to unstimulated MDDCs from the same young participants.

To gain a better understanding of the ability of eLNP treatment to initiate TGF-β production, PBMCs isolated from both healthy young and aged participants were treated with eLNP for either 6 or 24 hrs (Figure 7b). Although no significant differences were initially observed between stimulated and unstimulated cells after 6 hrs in TGF-β1 and TGF-β2, TGF-β3 levels were significantly higher in both young (p p<0.0001) and older participants (p p<0.0001). TGF-β1 (young, p=0.0034; old, p<0.0001) and TGF-β3 (young, p=0.0004; old, p=0.0022) showed significant expression in both young and older adults after 24 hrs stimulation with eLNP Importantly, other than TGF-β2 at 24-hour stimulation (p=0.0737), TGF-β1 production in young participants was reduced compared to old at 24 hrs (p=0.0047) (Figure 7b). Altogether, these data suggest that eLNP treatment is a potent inducer of TGF-β production in PBMCs while also suggesting that there are age-associated differences in the ability of eLNP to stimulate TGF-β production in young vs aged healthy adults. The ability of eLNP to induce TGF-β production and the differences observed between young and aged adults in the level of TGF-β that is induced are likely of importance, particularly in the use of eLNP during vaccination, due to the essential roles that TGF-β plays in the differentiation and function of T cells.

## Discussion

Aging is associated with increased morbidity and mortality to viral infections including SARS-CoV-2^31^. Of the multiple factors that contribute to this, impaired vaccine responsiveness and the creation of immunological memory are key contributors. To continue to identify vaccine platforms and adjuvants that strongly promote immune responses in older adults is critically important. Recently, modified nucleoside mRNA vaccines were approved to control the ongoing pandemic caused by SARS-CoV-2 (COVID-19). These vaccines utilize LNP containing ionizable lipids, that have been shown to potently induce IL-6 secretion and T_FH_ and GC B cell generation in humans^1, 32, 33, 34^. It is worth noting that while LNP have been used in many different mRNA vaccine platforms, several studies have investigated the adjuvant effect. However, differences in LNP formulations and study design make it difficult to compare and correlate findings. As more mRNA-LNP vaccines are being designed for other infectious diseases, studies investigating the mechanism of immune modulation including the effect of LNP on innate immunity are critical to improve the efficacy of vaccines in older populations.

In this study, we expand upon the mechanism of ionizable eLNP formulation that Alameh et. al. previously investigated. It was previously reported that the adjuvant properties of this LNP formulation are linked to the ionizable lipid component of LNP and that there is an intrinsic ability of the eLNP to promote IL-6 secretion in mice and subsequent T_FH_ induction. We show that eLNP formulation induces the maturation and activation of DCs and monocytes as measured by the frequency of co-stimulatory surface receptors and the production of cytokines and chemokines (Fig. 1). We also show that there are age-specific differences in the maturation and activation in both MDDCs and subsets from PBMCs, namely cDC1, cDC2, and CD14^dim^ CD16^+^ monocytes (Fig. 5). This suggests the possibility that eLNP may mediate its effect on germinal center formation and T_FH_ function at least in part, by altering the maturation status of antigen- presenting cells^1^. While Alameh et al show that the adjuvant effect of eLNP is maintained in MyD88-and MAVS-deficient mice, in human PBMCs, we show that eLNP treatment can induce the phosphorylation of IRF7 and TBK-1 (Fig. 2), key signaling molecules in antiviral signaling pathways potentially indicating signaling endosomally through other TLRs (e.g., TLR2, TLR3 and TLR4). TBK-1 induces innate antiviral type I IFNs but also plays a much broader role in antibody formation and autophagy^35, 36^. More recent studies have shown that TBK-1 associates with ICOS and plays a role in the differentiation of GC T_FH_ cells and the development of B cell responses^37^. Consequences of decreased TBK-1 activity in cDC2 due to aging in response to eLNP (Fig. 2b) may curtail humoral immunity through this ICOS-driven GC pathway. In fact, it has also been demonstrated that TBK-1 signaling is, at least partially, responsible for mediating the adjuvant effect of DNA vaccines by differentially controlling DNA-activated innate immune signaling^38^. Of note, one study recently done by Takanohashi et al, 2022 looked at interferon stimulated genes (ISG) in response to eLNP transfected whole blood^39^. Upon 6 hr eLNP transfection, this group did not see an elevated ISG score nor an induction of TBK-1 transcripts in healthy controls. This difference in results obtained could be due to several factors: 1) whole blood vs the use of isolated PBMCs and purified DCs as the cell populations of interest in our study are infrequent in whole blood; 2) our study looked at phosphorylation of IRF7 and TBK-1 and protein levels of cytokine and chemokine and not on transcript expression.

Importantly, we have found that in response to eLNP treatment, cDC2, cDC1, and CD14^dim^ CD16^+^ monocytes from older donors have upregulated PD-L1 when compared to younger counterparts (Fig. 5c). PD-L1, through virtue of its function, regulates the germinal center response by limiting T_FH_ differentiation and function^40^. However, the upregulation of PD-L1 on cDCs is a critical role to protect against killing of cytotoxic T lymphocytes (CTLs), errant PD-L1 expression might contribute to the decrease in immune responsiveness in older adults.

To investigate the mechanism of eLNP as a regulator of T_FH_ differentiation and innate immunity, we evaluated TGF-β production. TGF-β is a potent modulator of proliferation, differentiation, and function of all of lymphocytes, dendritic cells, and macrophages, essentially regulating both innate and antigen-specific immunity. In monocytes and DCs, TGF-β1 is mostly suppressive through inhibition of cell proliferation and reduction of reactive oxygen and nitrogen species^41^. TGF-β1 also acts as a chemotactic factor and can induce migration in monocytes and can suppress the type I IFN response in alveolar macrophages^42, 43^. We show that TGF-βexpression was significantly increased following eLNP stimulation in PBMCs from older individuals. In contrast, TGF-β expression in MDDCs was reduced in younger individuals therefore maintain the imbalance between old vs young upon eLNP stimulation (Fig. 7a). However, at 24 hrs stimulation with eLNF, PBMCs from older adults significantly increase production of TGF-β1 when compared with their younger counterparts. Increased TGF-β1 production by these cells might play a role in the overall decreased antiviral and vaccination response. Coupled with our finding that the addition of eLNP significantly increases the ability of cDC2 and cDC1 from younger and older donors to phagocytose, there is an evident role of eLNP in modulating vaccine responsiveness. In fact, cDC2 show a significant decrease in phagocytic ability when stimulated with LPS/IFN-γ in older donors (Supplemental Fig. 6). That age-specific difference is rescued when stimulated with eLNP bringing levels of phagocytosis to that of younger donors indicating that eLNP can potentially be enhancing overall phagocytosis. TGF-β signals through STAT3:STAT4 to promote the differentiation of T_FH_ cells^16^ however the molecule also plays a role in the differentiation of T helper 17 (Th17) cells in inflammatory conditions in the presence of IL-6. We show an induction of IL-6 in PBMCs when stimulated with eLNP in both younger and older donors (Fig. 5) suggesting multiple roles of TGF-β under eLNP stimulation.

A recent study showed that after vaccination with two doses of Pfizer-BioNTech mRNA vaccine (BNT162b2), pSTAT 1 and pSTAT3 was increased as well as the anti-inflammatory cytokine IL-10 further confirming a role for eLNP in initiating these responses^44^. We also show that in response to eLNP IL-12 and IL-21 is secreted by MDDCs from older donors (Fig. 5b) and PBMCs from older and younger donors (Fig. 5d) suggesting a pro- T_FH_ inducing environment further indicating that eLNP is playing critical roles during vaccination^15^. We believe that our study provides important insight into the mechanism of action of eLNP such as those used in the Pfizer and Moderna COVID-19 vaccines.

## Experimental Methods

### Human Samples:

Blood samples were obtained from healthy donors at Martin Memorial Health Systems (Florida). Consenting adults were screened using a questionnaire determining their demographic information, medication usage, and comorbidities. Participants were excluded with any acquired immunodeficiency or immunomodulating medications (such as steroids or chemotherapy), pregnancy, history of cancer, and history of cirrhosis or renal failure, or antibiotic use within 2 weeks of recruitment. Blood samples were taken from individuals aged 18–80 and were made into single cell PBMC suspensions and frozen in bovine serum albumin (Sigma) plus 10% DMSO (VWR) for cryopreservation in liquid nitrogen. The Institutional Review Boards at the relevant institutions approved all procedures, and all participants provided signed informed consent.

### Empty LNP generation

The LNP formulation used in this study is proprietary to Acuitas Therapeutics; the proprietary lipid and LNP composition are described in US patent US10,221,127. Briefly, an ethanolic lipid mixture of a proprietary ionizable lipid, DSPC, cholesterol, and polyethylene glycol-lipid was rapidly mixed with an acidic aqueous solution (PMID: 23799535^45^), dialyzed, concentrated, frozen and stored at −80 °C. Empty LNP were formulated at an equivalent mRNA concentration of 1 mg/ml and total lipid concentration of 30 mg/ml^11^. The mean hydrodynamic diameter of eLNP measured by dynamic light scattering using a Zetasizer nano ZS (Malvern) was ~80 nm with a polydispersity index of 0.02–0.06.

### Determining Optimal Dose of eLNP

PBMCs were stimulated with eLNP in a twofold dilution to determine optimal dose. Optimal concentrations of eLNP were selected based on median production of IFN-g and an 85% or more survival rate (Supplemental figure 7).

#### Generation of human monocyte derived dendritic cells (MDDCs):

Previously cryopreserved human PBMCs from young (<65yrs old) and aged (>65yrs old) donors were thawed in RPMI 1640 (Corning) supplemented with 10% fetal bovine serum and 1% penicillin/streptomycin. PBMCs were enriched for CD14+ CD16+ monocytes via negative selection using EasySep^™^ human monocyte enrichment kit without CD16 depletion (STEMCELL Technologies) according to the manufacturer’s protocol. Following enrichment, monocytes were counted and resuspended at a density of 2 × 10^6^ cells/mL in serum-free CellGenix^®^ GMP dendritic cell medium (CellGenix) supplemented with 100ng/mL of recombinant human GM-CSF (Gemini Bio-products) and 20ng/mL of recombinant human IL-4 (Gemini Bio-Products). 1mL of the resuspended cells was added in 24- well plates for a total of 1 × 10^6^ cells per well. Monocytes were differentiated for 48 hrs. prior to simulation.

### *In vitro* stimulation of MDDC

Monocytes were stimulated for 24hrs with 0.5μg/mL of LPS (Invivogen, Cat# TLRL-eblps) plus 40ng/mL of IFN-γ (Invivogen, Cat#300–134P) or 0.78μg of empty lipid nanoparticles (eLNP) in 1mL of GM-CSF and IL-4 supplemented medium. Unstimulated control cells were maintained in GM-CSF and IL-4 supplemented medium for 24hrs. Following stimulation, dendritic cells were harvested and analyzed via flow cytometry and supernatants were collected after stimulation and frozen at −80°C

#### Flow cytometric analysis of human MDDCs:

Briefly, after stimulation, harvested MDDCs were washed twice with fluorescence-activated cell sorting (FACS) buffer (PBS containing 2% FBS), surface stained using antibodies for 30 minutes in 100 ul of FACS buffer. LIVE/DEAD Fixable Dead Cell Stain (Life Technologies, Cat: L34957) was used to gate on live cells. Samples were acquired on a BD^™^ LTR Fortessa (BD Biosciences), and analysis was conducted using FlowJo software (version 10). When gating, doublet cells were excluded and MDDCs were gated on live CD3^−^ CD19^−^ CD56^−^ CD11c^+^ cells.

#### *In vitro* stimulation of monocyte and DC subsets:

PBMCs from healthy young and older donors were plated at a volume of 1.0 × 10^6^ cells per well in a round 96-well plate in a volume of 100 ul of complete RPMI medium (RPMI 1640 with L-glutamine [Corning Cellgro, Manassas, VA] supplemented with 10% FBS and 13 [50 U] penicillin-streptomycin [Invitrogen, Carlsbad, CA]). For experiments involving LPS/IFNg stimulation, PBMCs were stimulated for 6 or 24 hrs with 0.5ug/mL LPS (Invivogen, Cat# TLRL-eblps) plus 40 ng/mL IFNg (Invivogen, Cat#300–134P). For experiments involving empty LNP PBMCs were stimulated for 6 or 24 hrs with 0.78 ug/mL eLNP Following stimulation, PBMCs were analyzed via flow cytometry and supernatants were collected after stimulation and frozen at −80°C

### *In vitro* stimulation of isolated monocytes

Monocytes were isolated from PBMCs with the StemCell Technology monocyte negative selection kit (Cat# 19359). Isolated monocytes were stimulated for 24hrs with 0.5μg/mL of LPS (Invivogen, Cat# TLRL-eblps) plus 40ng/mL of IFN-γ (Invivogen, Cat#300–134P) or 0.78μg of empty lipid nanoparticles (eLNP) in 1 mL of GM-CSF and IL-4 supplemented medium. Unstimulated control cells were maintained in GM-CSF and IL-4 supplemented medium for 24hrs. Following stimulation, dendritic cells were harvested and analyzed via flow cytometry and supernatants were collected after stimulation and frozen at −80°C

#### Phosflow cytometry analysis of monocyte and DC subsets and costimulatory markers:

The stimulated PBMCs from adult or older adult donors were prepared and incubated with fluorochrome-conjugated antibodies for flow cytometry. Briefly, after stimulation, cells were washed twice with fluorescence-activated cell sorting (FACS) buffer (PBS containing 2% FBS), surface stained using antibodies for 30 minutes in 100 ul of FACS buffer, permeabilized using 300 μl of cold BD phosflow buffer III (BD Biosciences) according to manufacturer’s instructions, intracellular phosphoprotein stained using antibodies against intracellular phosphorylated IRF7(pS477/ pS479, BD Biosciences), pTBK-1 (BD Biosciences, or STING (BD Biosciences in 50 ul FACS buffer for 1 hour, and then fixed using 2% PFA for 15 minutes at 37°C. LIVE/DEAD Fixable Dead Cell Stain (Life Technologies, Cat: L34957) was used to gate on live cells. Samples were acquired on a BD^™^ LTR Fortessa (BD Biosciences), and analysis was conducted using FlowJo software (version 10). Cells were phenotyped as follows: cDC2 were Lineage− (CD19− CD3− CD56−CD20−) HLA-DR+ CD11c+ CD1c+ CD141− CD303−, cDC1 were Lineage− HLA-DR+ CD11c− CD1c− CD141+ CD303^−^, pDC were Lineage− HLA-DR+ CD11c− CD1c− CD141− CD303+, Classical monocytes were Lineage− HLA-DR+ CD14+CD16−, Intermediate monocytes were Lineage^−^ HLA-DR^+^ CD14^+^ CD16^+^, and non-classical monocytes were Lineage^−^ HLA-DR^+^ CD14^dim^ CD16^+^.

#### *In vitro* phagocytosis assay:

PBMCs from healthy young and older donors were plated at a volume of 1.0 × 10^6^ cells per well in a round 96-well plate in a volume of 100 μl of complete RPMI medium at 37°C and at 4°C as a negative control. PBMCs were then stimulated as stated above for 24 hrs. After a 24 hour stimulation period, cells were washed twice with FACS buffer to remove agonist and incubated with 0.04 μm fluorescent microspheres (Invitrogen, Cat: F8794) for 3 hrs. The cells were then washed twice with FACS buffer to remove any beads from the outside of the cell and prepared for flow cytometry.

#### Phagocytic flow cytometry analysis of monocyte and DC subsets:

The PBMCs from adult or older adult donors were prepared and incubated with fluorochrome-conjugated antibodies for flow cytometry. Briefly, after stimulation, cells were washed twice with FACS buffer, surface stained using antibodies for 30 minutes in 100 μl of FACS buffer, and then fixed using 2% PFA for 15 minutes at 37°C. Cells were phenotyped as stated above.

#### Cytokine and chemokine analysis:

Supernatants collected from PBMCs during stimulation were analyzed for chemokine/cytokine levels using the human immune monitoring 65-Plex ProcartaPlex^™^ Panel (Invitrogen^™^). This kit was used to determine the levels of 65 cytokines, chemokines, growth factors, and soluble receptors produced by MDDCs, 24 hrs after stimulations, and PBMCs 6 and 24 hrs after stimulations. The following human chemokine/cytokine premixed panel was used according to the manufacturer’s protocol: G-CSF (CSF-3), GM-CSF, IFN alpha, IFN-g, IL-1 a, IL-1 b, IL-2, IL-3, IL-4, IL-5, IL-6, IL-7, IL-8 (CXCL8), IL-9, IL-10, IL-12p70, IL-13, IL-15, IL-16, IL-17A (CTLA-8), IL-18, IL-20, IL-21, IL-22, IL-23, IL-27, IL-31, LIF, M-CSF, MIF, TNF-a, TNF-b, TSLP BLC (CXCL13), ENA-78 (CXCL5), Eotaxin (CCL11), Eotaxin-2 (CCL24), Eotaxin-3 (CCL26), Fractalkine (CX3CL1), Gro-alpha (CXCL1), IP-10 (CXCL10), I-TAC (CXCL11), MCP-1 (CCL2), MCP-2 (CCL8), MCP-3 (CCL7), MDC (CCL22), MIG (CXCL9), MIP-1a (CCL3), MIP-1b (CCL4), MIP-3a (CCL20), SDF-1a (CXCL12), FGF-2, HGF, MMP-1, NGF-b, SCF, VEGF-A, APRIL, BAFF, CD30, CD40L (CD154), IL-2R (CD25), TNF-RII, TRAIL (CD253), TWEAK. Data was acquired on a Luminex^™^ FLEXMAP 3D^™^ System using bead regions defined in the protocol and analyzed using Belysa Curve Fitting Software (Sigma Aldrich). Standard curves were generated, and sample concentrations were calculated in pg/mL.

#### Statistics:

All flow cytometry, Luminex, and confocal data were analyzed using GraphPad Prism v9. Where appropriate, stimulations were subtracted from their background controls, i.e., LPS/IFNg stimulated cells were subtracted from unstimulated, RIG-I agonist was subtracted from LyoVec only control, and G10 STING agonist was subtracted from a DMSO control. Unpaired, non-parametric Mann Whitney test was used when comparing two groups. The Paired multiple t-test and non-parametric one-way ANOVA (Friedman) test was used when comparing more than two groups to each other. (*p < 0.05, **p < 0.01, ***p < 0.001, ****p < 0.0001).
